# Modeling the Wildlife–Livestock Interface of Cattle Fever Ticks in the Southern United States

**DOI:** 10.3390/insects16090940

**Published:** 2025-09-06

**Authors:** Vera W. Pfeiffer, José-María García-Carrasco, David W. Crowder, Massaro W. Ueti, Karen C. Poh, Javier Gutierrez Illán

**Affiliations:** 1 Department of Veterinary Microbiology and Pathology, Washington State University, Pullman, WA 99164, USA; vera.pfeiffer@wsu.edu; 2 Department of Entomology, Washington State University, Pullman, WA 99164, USA; j.garciacarrasco@wsu.edu (J.-M.G.-C.); dcrowder@wsu.edu (D.W.C.); javier.illan@wsu.edu (J.G.I.); 3 Animal Disease Research Unit, United States Department of Agriculture, Agricultural Research Service, Pullman, WA 99164, USA; massaro.ueti@usda.gov

**Keywords:** cattle fever ticks, habitat suitability models, cattle, white-tailed deer, nilgai

## Abstract

Cattle fever ticks, *Rhipicephalus microplus* and *Rhipicephalus annulatus*, transmit *Babesia* pathogens, the causative agents of cattle fever worldwide. Although eradicated from the United States, increasing incursions of cattle fever ticks in Texas have put considerable strain on the Cattle Fever Tick Eradication Program (CFTEP). The movement of ticks between wildlife and cattle along the Texas–Mexico border complicates control efforts. Here, we used habitat suitability models, the literature, and quantitative survey data to project the distributions of native and introduced ungulates in Texas. Specifically, we used habitat suitability models and downscaling to estimate potential overlap between cattle and free-ranging white-tailed deer (*Odocoileus virginianus*) and nilgai (*Boselaphus tragocamelus*) that may carry cattle fever ticks and generate maps of estimated tick exposure risk. Our findings suggest that the introduction and spread of exotic ungulates, such as the nilgai antelope, may facilitate the expansion of cattle fever ticks within and beyond the historical quarantine zone established in 1943. The increasing range of nilgai populations could enhance landscape connectivity for cattle fever ticks in sensitive areas along the Texas–Mexico border. By combining these models with cattle inventory data, we provide tools to help the CFTEP better allocate resources, monitor tick populations, prevent incursions, and implement early interventions.

## Simple Summary

Cattle fever is a serious disease caused by microscopic parasites spread by ticks. Although the disease was eliminated from the United States decades ago, these ticks continue to reappear in Texas. One major challenge is that the ticks can move between cattle and wild animals, especially along the Texas–Mexico border. In this study, we used maps, wildlife data, and modeling tools to estimate where domestic and wild animals, such as white-tailed deer and the non-native nilgai antelope, are likely to be present. These animals can carry ticks, making it challenging to control their spread. We found that nilgai, which have been expanding their range in Texas, may help ticks move across the landscape, including into areas where they were previously controlled. We created maps showing where the risk of tick spread is highest. This information can help officials focus control efforts in key areas, better protect cattle, and stop the disease from returning to the United States. Our work is useful in protecting livestock health and the cattle industry.

## 1. Introduction

The tick species *Rhipicephalus microplus* and *Rhipicephalus annulatus* are major cattle pests globally as they can transmit the causative agents of bovine babesiosis (*Babesia bovis* and *Babesia bigemina*), commonly known as cattle fever [1,2,3]. Cattle fever disease was highly damaging to the cattle livestock industry in the southern United States for much of the early 20th century until the creation of the Cattle Fever Tick Eradication Program (CFTEP), which led to the eradication of cattle fever ticks and bovine babesiosis from the United States. Although cattle fever ticks are largely considered eradicated from the United Sates, both tick species continue to reappear along the Texas–Mexico border, known as the Permanent Quarantine Zone (PQZ) [4,5]. Yet, despite the intensive and persistent control efforts, cattle fever ticks are consistently found inside the Permanent Quarantine Zone due to extraneous circumstances such as cattle movement outside regulated protocols, stray livestock, and wildlife host movement.

The persistence of cattle fever ticks in the southern United States may be due in large part to their ability to use both managed and wildlife ungulate species as hosts, with the white-tailed deer (*Odocoileus virginianus*) considered the primary host for cattle fever ticks in the PQZ [5]. With habitat improvements, predator control, restocking, limits on hunting, and game ranching, populations of white-tailed deer in the southern United States have grown and have recently been estimated to exceed 5 million individuals [5,6,7]. Exotic wildlife species are also common in the quarantine zone and likely support cattle fever tick populations, with the nilgai (*Boselaphus tragocamelus*), an introduced species from India, serving as a major host that has complicated eradication efforts in the quarantine zone [4,5,6,8,9]. The presence of native and exotic wildlife species poses threats to continued cattle fever tick control, as these ungulates can serve as hosts to sustain tick populations when managed livestock are not present [8,10,11,12].

Cattle fever ticks are one-host ticks, spending all three of their life stages on a single animal, approximately three weeks between attachment as a larval tick to a replete adult [13,14,15]. During this time, wild ungulate hosts can travel distances greater than 100 km, distributing ticks throughout the landscape as they move [16,17,18,19,20]. Given the limited availability of effective landscape-level measures to control cattle fever ticks on wildlife hosts, it is essential to know which areas are most appropriate for these hosts and ticks to generate more effective risk estimates. Prior modeling efforts have explored the relationship between distributions of white-tailed deer and cattle and predicted that managing large vegetation patches may aid in the control of tick-borne pathogens, as these areas attract more animals [21]. However, similar efforts to model the relationships in the cattle fever tick system involving cattle and native and exotic wildlife hosts such as nilgai in the southern United States and northern Mexico have not been conducted.

Here, we conducted a study to assess how the distribution of wildlife hosts affects the risk of cattle fever tick outbreaks. Our study had three complementary objectives. First, we predicted areas of suitable habitats for white-tailed deer and nilgai in Texas and surrounding landscapes. Second, we identified areas of the landscape where managed cattle populations overlapped with potential wildlife hosts of cattle fever ticks, indicating areas of potential tick spread. Third, we calculated an index of shared habitat use intensity between cattle and free-ranging ungulates, which indicates the potential for the acquisition of cattle fever ticks from these potential wildlife hosts. Exploring the distribution and potential range expansion of existing wildlife hosts for cattle fever ticks and overlaying these models with cattle inventory will provide predictions that can be used to guide tick monitoring, prevent incursions, and enable early interventions for tick species that represent a continuing threat to cattle production.

## 2. Materials and Methods

### 2.1. Study Area and Species Occurrence Data

Our study considered habitat overlap between managed cattle and wildlife hosts of cattle fever ticks across Texas, including southern Texas where the PQZ is established [22]. Texas is located in the southern United States, with a dramatic moisture cline that extends from the Gulf Coast inland to the Chihuahuan Desert. This precipitation gradient likely limits the distribution of cattle fever ticks to the eastern and southern parts of the state due to the desiccation risk they face in the dry western and northern regions [23]. Cattle ranching occurs across the state, though more feedlots are located in the northern reaches, and more pasture-fed cattle are found in the eastern and southern parts of the state [24].

To build habitat suitability models, we gathered population density and occurrence data for cattle, white-tailed deer, and nilgai. Data for total cattle including calves from each county in Texas on 1 January 2022 were used, with the number of cattle on feedlots removed to focus on cattle in pasture [24]. Occurrence data for white-tailed deer and nilgai hosts were obtained from public databases using R statistical software version 4.2.3 (R Foundation for Statistical Computing, Vienna, Austria) [25] and the package *spocc*, and all data were accessed in the Global Biodiversity Information Facility database [26]. To ensure good coverage for each of the host species, we extracted 10,000 random records for white-tailed deer in Texas and nilgai in India. A habitat suitability model for nilgai was fit across their native range of northern India in order to project their habitat suitability from a more stable distribution onto the Texas landscape based on the same climatic and landscape predictor variables. Records were filtered to remove duplicate records as well as points that were located in urban or managed areas (e.g., museums and zoos). As occurrence data in public databases are often biased, with more observations near population centers [27], we thinned the data to include at most one observation within each grid cell, using a 20 km resolution for nilgai and 50 km for white-tailed deer [28]. After filtering records, we had 6979 records for white-tailed deer and 794 records for nilgai.

### 2.2. Explanatory Variables for Habitat Suitability Models

We considered the effects of 19 environmental, topographic, and landscape variables on the distribution of each ungulate host species (Table 1). These environmental variables were aligned with ungulate host records within Google Earth Engine (Google LLC, Mountain View, CA), a web-based platform that makes available satellite imagery and other spatial datasets [28,29]. Climate variables were extracted from the Chelsa dataset and Moderate Resolution Imaging Spectroradiometer (MODIS) imagery [30]. The variables diurnal temperature range, maximum temperature, minimum temperature, annual precipitation, and precipitation seasonality were gathered from these datasets [30]. Land surface temperature was accessed to extract the number of days below 0 °C and below −3 °C [31]. These variables were included because near-complete mortality for nilgai have been reported in Texas at these temperatures and, thus, we considered areas with these conditions to be unlikely to support nilgai range expansion [32].

Topographic variables included in the habitat suitability models included elevation and slope, which were extracted from NASA’s Shuttle Radar Tomography Mission-derived digital elevation model [33]. In this model, a global ALOS multi-scale topographic position index distinguishes ridges from valleys at multiple scales, allowing for the precise calculation of elevation. The metric uses moving windows of multiple scales to calculate the difference between local and average regional elevation. The continuous heat-insolation load index (CHILI) was also included to represent the effect of topographic shading on evapotranspiration. This value was calculated based on sun altitude equivalent to equinox in early afternoon. These metrics were developed by Conservation Science Partners as ecologically relevant classifications of landforms for climate adaptation planning and were accessed on the Google Earth Engine web platform [34].

Reflectance-based land cover variables included the normalized difference vegetation index (NDVI), normalized difference built-up index (NDBI), and modified normalized difference water index (MNDWI). These metrics were calculated from the European Space Agency’s Sentinel 2 multispectral satellite imagery: (i) *NDVI = (NIR − RED)*/*(NIR + RED)*; (ii) *NDBI* = *(SWIR1* − *NIR)/(SWIR1 + NIR)*; and (iii) *MNDWI = (GREEN − SWIR1)/(GREEN + SWIR1)*, with *NIR* defined as “near infrared” and *SWIR1* defined as “short-wave infrared band 1.”

Four additional land cover and configuration variables were included in the habitat suitability models. These variables included the percent cover of short vegetation (0–100%) in grasslands, where non-grassland cover types were allocated a 0 value based on Global Land Analysis and Discovery (GLAD) program products at a 100 m resolution [35]. Second, the area of land of forest/short vegetation edge within 1 km was estimated by building a layer that combined grassland and cropland then buffering the combined dataset to expand the layer by 1 km using the buffer tool in ArcGIS 10.8.2 (Esri, Redlands, California) and applying the same buffering procedure to the forest layer to expand it by 1 km. The area of this edge class was calculated within a 1 km moving window to create a surface representing the proportion of forest/short vegetation edge within 1 km. Third, a layer representing distance to open water or a wetland, up to a maximum of 50 km, was also included in the models. Fourth, percent tree cover was accessed as a preprocessed layer based on NDVI calculated from MODIS imagery at a 250 m resolution. Tree canopy was characterized by the NDVI value, and median percent canopy cover from 2020 was extracted at a 1 km grid cell resolution [36].

Finally, the 2020 human population density was included as a land use variable accessed as GPWv411: Population Density (Gridded Population of the World Version 4.11)—band 5 from NASA SEDAC at the Center for International Earth Science Information Network [37]. The 1 km resolution surface represents the number of people per sq km, distributed based on the proportional allocation of the population assessed in censuses and by administrative districts. The data were extrapolated from available census data based on demographic models.

### 2.3. Habitat Suitability Models

We used occurrence data for ungulates as well as the environmental data to build habitat suitability models for each species. We used random forests, which is an ensemble modeling technique comprised of many decision trees that output the majority vote of the individual trees for each model run, combining bagging with the random selection of features [38]. Random forest models assessed the relationship between the 19 environmental, topographic, and landscape variables (Table 1) and the occurrence or density data for each ungulate species. For each model run, 500 trees were fitted based on five randomly selected explanatory variables per split with a minimum of 10 leaves and no maximum limit of leaves. The fraction of input to bag per tree was 0.5 (default), and the resulting ungulate distribution surfaces map the suitability of the habitat for the species on a scale from 0 to 1. The model also calculates variable importance, which provides a metric of the sensitivity of the model results to the inclusion or removal of each variable.

Since only presence data were available for white-tailed deer and nilgai, random pseudo-absence points were generated to fit the models. Both species, the white-tailed deer and nilgai, are highly mobile with dispersal distances over 10 km and have well-established populations across their ancestral ranges [16,17,18,19]. We therefore resampled the pseudo-absence points to match the number of thinned presence points with each model before model validation was performed.

### 2.4. Model Validation

Multiple-block cross-validation was used to partition data for model training and validation for 10 model runs using 70-30 splits. We applied a spatial-block cross-validation to partition the data into model training and model validation datasets for each model run [39,40]. Spatial-block partitioning ameliorates undesirable effects of autocorrelation and reduces the inflation of accuracy metrics compared to random partitioning [39]. The random block splits generated during each iteration created different training and validation datasets. Pseudo-absences were randomly generated within each set of training and validation blocks for each model run [41]. The same number of pseudo-absences as presence data was created for nilgai, resulting in a completely balanced dataset that has been argued to perform better with machine-learning techniques; a somewhat imbalanced dataset was used for white-tailed deer, but this was minimized by increased thinning of the data at a 50 km scale instead of a 20 km scale [42,43,44].

Model accuracy was assessed on the validation partition of each iteration of model fitting using threshold-independent area under the curve of the receiver operator characteristic curve (AUC-ROC) [45] and the area under the precision–recall curve (AUC-PR) [46]. The AUC-ROC values can range from 0 to 1, where 1 indicates perfect discrimination between presence and absence–pseudo-absence locations and a value of 0.5 discrimination is no better than random. The AUC-PR metric also ranges from 0 to 1, where 1 is perfect prediction of habitat suitability and random performance is equal to the prevalence of presence locations in the dataset [46].

After the initial habitat suitability models were fit and validated, the top predictors based on variable importance from the random forest model for each ungulate host were retained in a final habitat suitability model. For the white-tailed deer, the final model included the variables diurnal temperature range, annual precipitation, elevation, NDVI, percent tree canopy cover, amount of forest/grassland edge, and distance to water. For the nilgai, the final model included the variables diurnal temperature range, minimum temperature, annual precipitation, precipitation seasonality, slope, and CHILI.

### 2.5. Assessing Overlap Between Wildlife Hosts and Cattle

Transmission of the *Babesia* spp. parasites that cause cattle fever requires habitats that support hosts, ticks, and pathogens. We thus constructed downscaled estimates of white-tailed deer, nilgai, and cattle hosts by using the output of the habitat suitability models as weighted probability surfaces to distribute quantitative population estimates. For white-tailed deer, we integrated quantitative population estimates from the Texas Parks and Wildlife Department based on surveys from 2020 and 2021 [47,48] into our habitat suitability models by distributing the regional estimate of deer as random points across the distribution model in a weighted raster surface using the *raster* package in R [49]. In this method, more points are allocated to cells that have higher habitat suitability values.

For nilgai, we downscaled density using habitat suitability models in a similar manner as with white-tailed deer but used three different scenarios, as rigorous estimates of nilgai population sizes are not available. Nilgai were first introduced to the Texas Gulf Coast in the 1920s and by 1971 had spread to nine counties [50,51]. By 1992, the population was estimated at >37,000 individuals, and current estimates of between 35,000 and 100,000 individuals make nilgai one of the most common ungulates in the state [5,6,8]. We estimated the wild nilgai population as having (i) 0, (ii) 30,000, or (iii) 100,000 individuals, allowing us to model scenarios without nilgai as well as the low and high end of current population estimates. In scenarios with nilgai, the individuals were added randomly to the southern Texas counties in the Lower Rio Grande Valley (Cameron, Hidalgo, and Willacy) and along the Coastal Bend (Brooks, Kenedy, Nueces, Kleberg, and San Patricio) based on the habitat suitability model.

**Figure 1:**
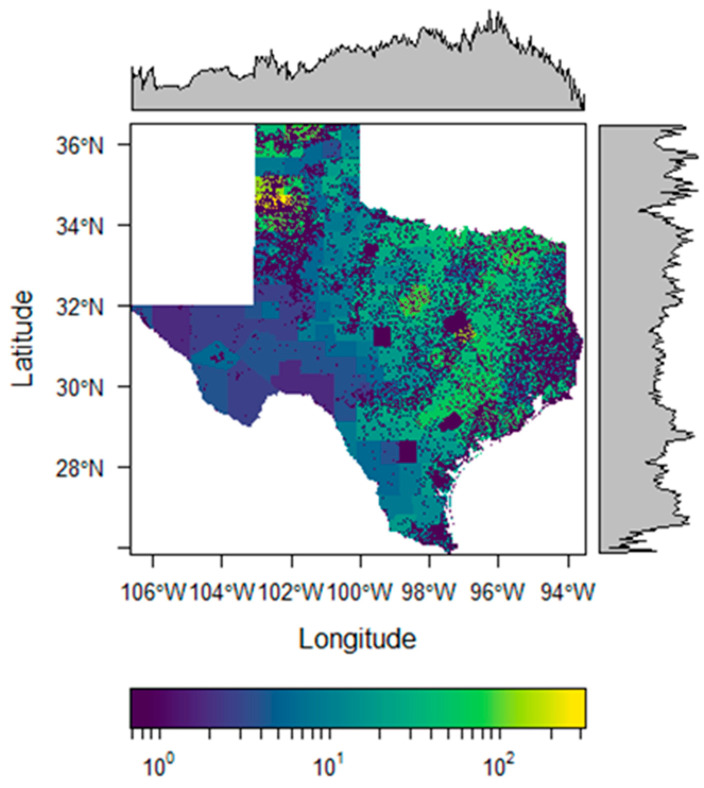
The density surface of pasture cattle. The color represents the estimated number of cattle per sq km, while edge plots symbolize the mean value of pixels in two-dimensional space.

To estimate the distribution of cattle, data for total cattle including calves from each county in Texas pasture fields on 1 January 2022 were used [24]. We then estimated how these cattle were distributed within counties by using land cover data to reclassify a bivariate (0 or 1) surface of potential cattle land (e.g., pasture, grassland, shrubland, barren, forest, deciduous forest, mixed forest, developed/open space, and developed/low intensity). Except for the brown dog tick (*Rhipicephalus sanguineus*) and the lone star tick (*Amblyomma americanum*), ticks generally do not thrive in feedlots or crops, so we eliminated croplands and developed lands [52,53]. The area of this potential cattle land was tallied at the county level, and the total number of cattle including calves was divided by the area of potential cattle land in square kilometers to estimate the cattle density for each county at a 1 km grid cell resolution. The calculated cattle density value was then multiplied by the bivariate (0 or 1) 1 km resolution cattle land surface to represent the estimated cattle density across the study extent, and these values were then plotted spatially with the package *RasterVis* in R [49] (Figure 1).

After all species densities were downscaled and estimated at a granular scale, the density estimates of white-tailed deer and nilgai (scenario with 30,000 individuals) were overlayed and summed to estimate the total density of ungulates per sq km across Texas. This total wild host ungulate density surface was multiplied with the pasture cattle density per sq km surface then normalized between 0 and 1. This created a relative index of overlap between pasture cattle and wildlife hosts of cattle fever ticks, representing an estimate of tick exposure risk.

## 3. Results

**Figure 2:**
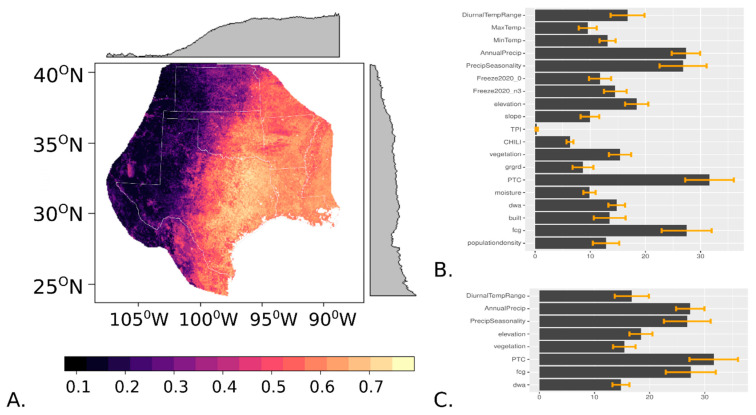
(**A**) Habitat suitability predictions for white-tailed deer in Texas and the surrounding region, with lighter colors representing higher suitability. Gray edge plots symbolize the mean value of pixels in two-spatial dimensions. The right panels exhibit the variable importance of each explanatory variable in the (**B**) random forest and (**C**) downscaled distribution models, with error bars explaining the standard deviation in variable importance across 10 model runs.

The habitat suitability for white-tailed deer was estimated to be highest across eastern and southern Texas, tapering off with lower values towards the western part of the study extent (Figure 2A). The arid conditions that limit the white-tailed deer distribution in the western part of the distribution map continued south into Mexico, curving eastward south of the Texas–Mexico border to create a natural bottleneck in the connectivity of habitat for this wildlife host of cattle fever ticks (Figure 2A). The top four variables affecting the distribution of white-tailed deer were percent tree cover (variable importance 31.6), amount of forest/grassland edge (variable importance 27.5), annual precipitation (variable importance 27.4), and precipitation seasonality (variable importance 26.8) (Figure 2B,C). The other four predictors retained for the final model were elevation (variable importance 18.4), diurnal temperature range (variable importance 16.8), NDVI (variable importance 15.4), and distance from water (variable importance 14.8) (Figure 2C). Across 10 model runs, the average AUC-ROC was 0.78, and the average AUC-PR was 0.75.

**Figure 3:**
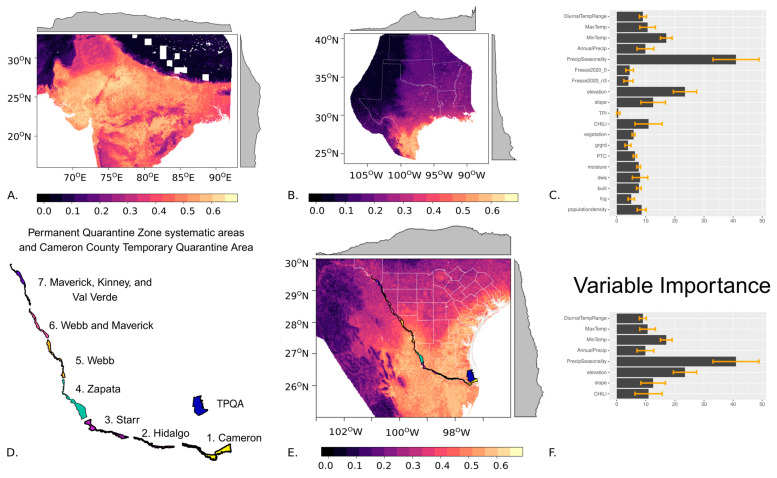
Habitat suitability predictions for nilgai in (**A**) India and (**B**) Texas and surrounding regions, with lighter colors representing higher suitability and gray edge plots symbolizing the mean pixel value. (**C**) Variable importance values for the random forest models, with error bars explaining the standard deviation across 10 model runs. (**D**) Systematic areas of the Permanent Quarantine Zone (PQZ) and the Temporary Preventative Quarantine Area (TPQA). (**E**) Fine-scale map of the nilgai habitat suitability model for South Texas including the PQZ and TPQA. (**F**) The variable importance in the downscaled distribution models, with error bars explaining the standard deviation across 10 model runs.

The habitat suitability model for nilgai that was fit in the species’ native range of northern and central India performed well, with an average AUC-ROC of 0.87 and AUC-PR of 0.81 across the 10 model runs (Figure 3A). The nilgai distribution model projected to Texas indicated a low probability of habitat suitability in the central and northern parts of the study extent and a high probability of habitat suitability in the southern regions (Figure 3B), including the eastern parts of the PQZ and temporary protective quarantine area (TPQA) along the Texas–Mexico border (Figure 3D,E). For nilgai, precipitation seasonality (variable importance 40.9), elevation (variable importance 23.4), and minimum temperature (variable importance 17.0) had the highest discriminatory power in the model (Figure 3C). Slope (variable importance 12.5), the continuous heat-insolation load index (variable importance 10.9), maximum temperature (variable importance 10.6), annual precipitation (variable importance 9.9), and diurnal temperature range (variable importance 9.0) were also included in the final model (Figure 3C,F).

**Figure 4:**
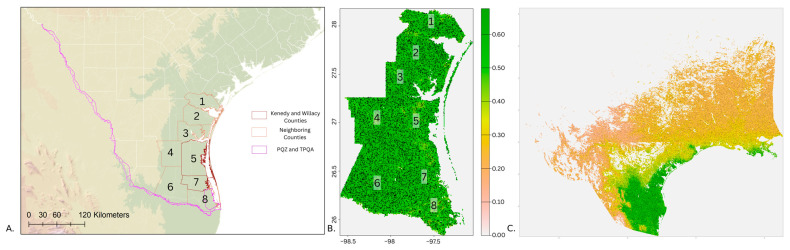
Nilgai population scenarios. (**A**) Texas counties in the Coastal Bend (1—San Patricio, 2—Nueces, 3—Kleberg, 4—Brooks, 5—Kenedy) and Lower Rio Grande Valley (6—Hidalgo, 7—Willacy, 8—Cameron) used in the scenarios, with nilgai currently primarily found in Kenedy and Willacy Counties (outlined in red). (**B**) Habitat suitability for nilgai in the Coastal Bend and Lower Rio Grande Valley in scenario 2 (30,000 nilgai added to the landscape). (**C**) The nilgai habitat suitability model considering the locations across Texas that remains above −3 °C, with nilgai applied from the scenario where 100,000 nilgai were added.

The three scenarios of nilgai population expansion led to higher densities in southern Texas, especially in the Lower Rio Grande Valley and Coastal Bend agricultural districts (Figure 4). Nilgai were predicted to increase in density by 24% in the Lower Rio Grande Valley and 50% in the Coastal Bend in the scenario where 30,000 nilgai were added and 5% and 27% in the scenarios where 100,000 nilgai were added, respectively (Figure 4). The Cameron County TPQA was estimated to be the most impacted part of the systematic areas for cattle fever tick monitoring based on expansion scenarios.

**Figure 5:**
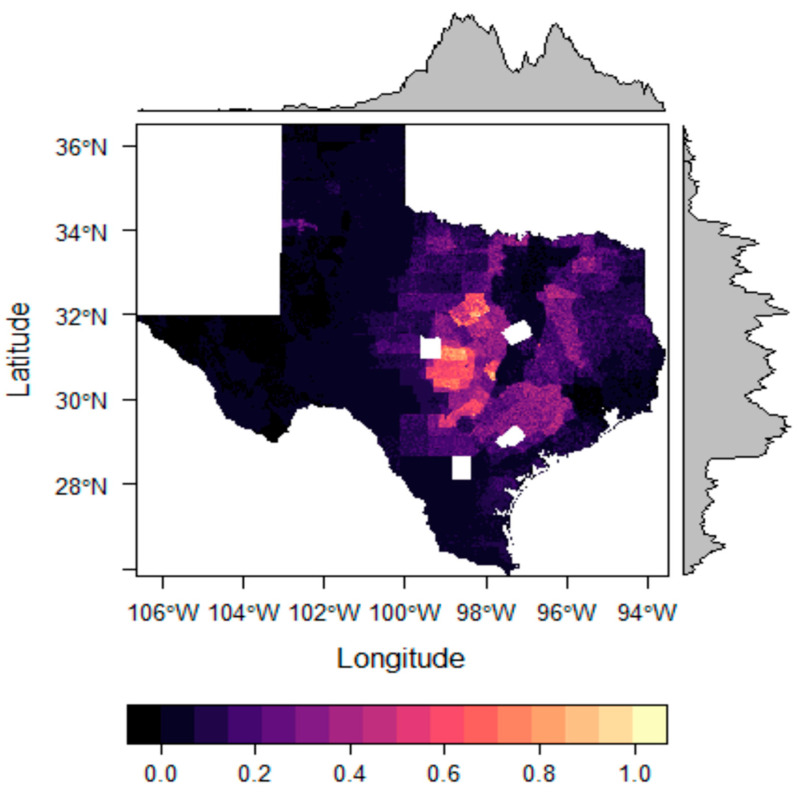
Relative shared habitat intensity of pasture cattle and wild ungulate hosts of cattle fever ticks, where the product of estimated pasture cattle/km^2^ and wild ungulate density/km^2^ (white-tailed deer and nilgai) was scaled between 0 and 1. The gray edge plots symbolize the mean value of pixels along two spatial dimensions; white portions of the study extent represent areas with low or no cattle inventory reported.

Although white-tailed deer and nilgai increases have been greatest in southern Texas, the highest predicted overlap of pasture cattle with free-range ungulates occurred in the Texas Hill Country in central Texas (Figure 5). Much of the western part of Texas had no estimated overlap suitable for cattle fever tick dispersal, as was the case for much of the Gulf Coast and a north–south strip in northeast Texas (Figure 5). In contrast, the central part of the state appeared to be highly suitable, with high cattle densities overlapping with suitable habitat for wildlife hosts that occur in high density (Figure 5).

## 4. Discussion

In this study, we projected and described downscaled distributions of white-tailed deer and nilgai antelope, which are two important hosts of cattle fever ticks in Texas, and estimated areas of likely shared overlap between these hosts and pasture cattle as a proxy for potential tick spread among the hosts. We also explored how three scenarios of nilgai absence or population expansion, which was modeled as variation in the number of nilgai individuals, could increase the connectivity of South Texas for cattle fever tick spread, especially around coastal reserves and through natural vegetation corridors. Our study provides further evidence that white-tailed deer hosts are one of the most common free-ranging ungulates in areas with cattle fever ticks [54]. Given that habitat suitability for nilgai was low in central Texas, it is likely that high populations of white-tailed deer in central Texas are the primary driver of the high overlap of pasture cattle and free-ranging ungulates. The prevalence of white-tailed deer hosts and the expansion of their populations over time has impeded cattle fever tick eradication in the past, prompting changes to policies on surveillance and tick control. Indeed, by 2004 infestations could be declared based on the presence of cattle fever ticks on wildlife hosts as well as in managed livestock, demonstrating that the prevalence of wild hosts is an effective predictor of infestation risk [6,12]. Our study provides insight into areas where the continued presence and increasing density of white-tailed deer in Texas highlight urgent control and monitoring.

Nilgai are well suited to the humid subtropical and hot semi-arid terrain that is common in southern Texas. Our habitat suitability models predict that the expansion of nilgai populations over the last century may increase the connectivity for cattle fever ticks around the southern border between Texas and Mexico. The first cattle fever tick infestation attributed to nilgai was reported in Cameron County Texas in 2007 [6]. Tick infestations that were attributed to nilgai increased in subsequent years, especially near Laguna Atascosa and the Lower Rio Grande Valley National Wildlife Refuge [6]. In 2016, exponential growth of nilgai populations in coastal Willacy County motivated a culling event of 210 nilgai, of which 192 individuals were found to be infested with cattle fever ticks, demonstrating their key role as a wildlife host that are commonly infested [6,8]. As with white-tailed deer, our study estimates habitat suitability and density of nilgai that can be used to guide future monitoring and control efforts to protect cattle.

Environmental change is a key factor that could affect the range expansion and spread of cattle fever ticks and their hosts [4,5,55]. Cold temperatures and drier conditions may limit nilgai [32] and cattle fever ticks, particularly *R. microplus* [13,56,57]. While the range of nilgai hosts is more restricted by cold temperatures and complex topography than white-tailed deer, their tolerance of hotter conditions has allowed them to spread further across South Texas and Mexico [32]. Warmer conditions could shift their distributions, enabling ticks and nilgai to establish in areas that were once considered inhospitable. Freezing temperatures in particular have been a key limiting factor for nilgai, as evidenced by mass die-offs during cold spells in Texas, which has restricted their northward movement [32]. While a cold temperature threshold was not included in our nilgai habitat suitability model based on data from India, it may be more relevant to the United States, where these temperatures occur.

As evidenced by scenarios depicted in this study, the addition of the nilgai distributions to the landscape along with native ungulates such as the white-tailed deer may increase the overlap between cattle populations and wildlife hosts of cattle fever ticks. This may be particularly true in sensitive, transitional environmental areas along the Texas–Mexico border [7,12]. The increased overlap in shared habitat between cattle and wildlife hosts could also mean increased infestation of cattle fever ticks across these different animal species [20,58]. For example, replete females can drop from a wildlife host that is passing through a pasture, and the resulting larvae can find a new host on cattle in the pasture, thereby perpetuating the life cycle of cattle fever ticks [12]. Alternatively, infested cattle may spread cattle fever ticks across the pasture, and wild ungulates can pick up ticks while passing through those pastures [12].

Wildlife hosts contribute strongly to cattle fever tick populations, making it necessary to investigate their roles in the range expansion of these vectors. The presence of multiple animal species within quarantine zones could also affect the spread of ticks and the pathogens they transmit. While this research focused on habitat suitability for white-tailed deer and nilgai, known cattle fever tick hosts, future research could assess the role of other wildlife hosts and incorporate animal movement data in modeling efforts to improve management and control. For example, high game fences along major roadways can reduce wildlife movement and limit cattle fever tick hosts if they are properly placed and maintained [7,18,19,20,50]. These strategies targeting cattle fever ticks at the livestock–wildlife interface will help direct efforts to monitor ticks, prevent incursions, and initiate early interventions to protect the livestock industry and associated ranchers in Texas and the southern United States.

## Figures and Tables

**Table 1:** Environmental variables and associated sources accessed in Google Earth Engine for habitat suitability models.

Variables	Source (Resolution)
***Environmental variables***	
Diurnal temperature range Max temperature	Chelsa BioClim+ (~1 km) Chelsa BioClim+ (~1 km)
Min temperature	Chelsa BioClim+ (~1 km)
Annual precipitation	Chelsa BioClim+ (~1 km)
Precipitation seasonality	Chelsa BioClim+ (~1 km)
Average annual number of days below 0 °C based on night land surface temperature, 2016–2020	MODIS Aqua Land Surface Temperature (daily, 1 km)
Average annual number of days below −3 °C based on night land surface temperature, 2016–2020	MODIS Aqua Land Surface Temperature (daily, 1 km)
***Topographic variables***	
Elevation	NASA SRTM DEM (~30 m)
Slope	NASA SRTM DEM (~30 m)
Multi-scale topographic position index (mTPI)	JAXA AVE (~270 m)
Topographic shading/heat insolation (CHILI)	JAXA AVE (~270 m)
***Land cover and configuration variables***	
Average vegetation/productivity (NDVI)	Sentinel 2 (weekly, 10 m)
Grassland cover gradient	GLAD project (100 m)
Percent tree canopy cover (PTC)	MODIS (250 m)
Moisture (MNDWI)	Sentinel 2 (weekly, 20 m)
Distance to water	GLAD project (100 m)
Normalized difference built-up index (NDBI)	Sentinel 2 (weekly, 20 m)
Forest and grassland or cropland edge	GLAD project (100 m)
***Land use variables***	
Human population density	NASA SEDAC

## Data Availability

Original data for white-tailed deer, nilgai, cattle, and their associated environmental factors are openly available in open-access databases, including the Global Biodiversity Information Facility (white-tailed deer and nilgai), USDA National Agricultural Statistics Service (cattle), and Google Earth Engine (environmental factors).
